# Acupuncture for the treatment of phantom limb syndrome in lower limb amputees: a randomised controlled feasibility study

**DOI:** 10.1186/s13063-016-1639-z

**Published:** 2016-10-25

**Authors:** Esmé G. Trevelyan, Warren A. Turner, Lynn Summerfield-Mann, Nicola Robinson

**Affiliations:** School of Health and Social Care, London South Bank University, 103 Borough Road, London, SE1 0AA UK

**Keywords:** Phantom limb, Randomised controlled trial, Acupuncture, Amputation, Mixed-methods research

## Abstract

**Background:**

Post amputation, the complication of phantom limb pain (PLP) is prevalent and difficult to manage. This study aimed to determine whether it was feasible and acceptable to undertake a definitive multicentred randomised controlled trial assessing the effectiveness of acupuncture for treating lower limb amputees with PLP.

**Methods:**

A mixed-methods embedded design, including a randomised controlled trial and semistructured interviews, was undertaken. A total of 15 participants with PLP were randomly assigned to receive either eight pragmatic Traditional Chinese Medicine acupuncture treatments and usual care or usual care alone over 4 weeks. Outcome measures were completed at baseline, weekly throughout the study and at 1 month post completion of the study and included: a numerical pain-rating scale, the Short-Form McGill Pain Questionnaire 2, the EQ-5D-5 L, the Hospital Anxiety and Depression Scale, the Perceived Stress Scale 10-item, the Insomnia Severity Index, and the Patient Global Impression of Change. Post completion of the trial, participants in the acupuncture group were interviewed about their experience. Feasibility-specific data were also collected.

**Results:**

Of 24 amputees meeting the study inclusion criteria, 15 agreed to participate (recruitment rate 62.50 %). Qualitatively, acupuncture was perceived to be beneficial and effective. Quantitatively, acupuncture demonstrated clinically meaningful change in average pain intensity (raw change = 2.69) and worst pain intensity (raw change = 4.00). Feasibility-specific data identified that before undertaking a definitive trial, recruitment, practitioner adherence to the acupuncture protocol, completion of outcome measures at 1 month follow-up and blinding should be addressed. Appropriate outcome measures were identified for use in a definitive trial. Data were generated for future sample size calculations (effect size 0.64). Allowing for a 20 % dropout rate, a sample size of 85 participants per group would be needed in a future definitive trial.

**Conclusions:**

A future definitive trial may be possible if the areas identified in this study are addressed. As acupuncture may be effective at treating PLP, and as this feasibility study suggests that a definitive trial may be possible, a multicentred trial with adequate sample size and blinding is now needed.

**Trial registration:**

ClinicalTrials.gov Identifier: NCT02126436, registered on 4 September 2014.

**Electronic supplementary material:**

The online version of this article (doi:10.1186/s13063-016-1639-z) contains supplementary material, which is available to authorized users.

## Background

Phantom limb pain (PLP) is defined as painful sensations in the missing portion of the amputated limb. It is neuropathic in nature and caused by a lesion of the somatosensory nervous system [1]. It may be chronic and has been found to influence individuals’ subjective wellbeing, affecting both physical and mental components of quality of life [2].

Currently, PLP is not well-managed. A systematic review evaluating the use of preemptive analgesia found that only one case-controlled study supported using combined bupivacaine, diamorphine, and clonidine. Epidural and perineural infusions containing local anaesthetic with or without opiates were deemed only effective for treating acute perioperative pain [3]. A small randomised controlled trial found that intravenous ketamine could significantly reduce PLP during, and for 30 mins after, infusion [4]. However, a subsequent systematic review found it to be ineffective [5]. The most commonly used first-line treatment is gabapentin [6] but a systematic review found this to be beneficial for short-term analgesic efficacy only [7]. Many case studies report positively on the effectiveness of mirror therapy [8] but few randomised controlled trials have been completed and adverse effects have been reported.

Acupuncture has been found to be effective for treating a variety of chronic pain conditions [9] but little quality evidence is available on the use of acupuncture for PLP. A recent systematic review identified only two nonrandomised controlled trials [10] and 26 case studies [11]. Further research is needed to evaluate the effectiveness of acupuncture for treating PLP, but prior to a definitive trial a study is needed to inform on feasibility.

This study aimed to evaluate the feasibility and acceptability of completing a small randomised controlled trial in preparation for a definitive multicentred randomised controlled trial [12]. Objectives were to: (1) explore the feasibility of recruiting, randomising, and retaining participants, (2) evaluate the feasibility and acceptability of having a usual care control, (3) evaluate adherence/compliance and acceptability of acupuncture as an intervention, (4) evaluate the appropriateness of outcome measures, (5) identify appropriate primary and secondary outcome measures which could be used in future trials, (6) explore the perceived effectiveness of acupuncture for treating PLP, (7) generate data on effect size for use in future sample size calculations, and (8) inform the development of an appropriate and feasible protocol for use in a definitive multicentred randomised controlled trial.

## Methods

A comparative effectiveness study using a mixed-methods embedded design, including a small randomised controlled trial incorporating semistructured interviews, was undertaken. The randomised controlled trial was unstratified, open, pragmatic, with two parallel arms, balanced randomisation and a usual care control. Interviews were cross-sectional. The study protocol has been published [12]. The trial was registered with ClinicalTrials.gov (NCT02126436). A Consolidated Standards of Reporting Trials (CONSORT) checklist is included within the article’s additional files (Additional file 1, CONSORT 2010 checklist of information to include when reporting a randomised trial.pdf). Ethical approval was granted from the NRES Committee London – Bloomsbury (14/LO/0817) and London South Bank University; the trial commenced in October 2014 and closed 1 year later in October 2015.

Participants were recruited from an NHS inpatient amputee rehabilitation unit in London. All participants were provided with information and were required to consent orally and in writing. Participants were included if they were: (1) 18 years of age or above, (2) had full cognitive ability and were able to communicate in English, (3) had had traumatic or medical amputation of a lower limb (more than just toes), and (4) were currently experiencing worst PLP of ≥5 on an 11-point verbal rating scale. Participants were excluded if they: (1) had congenital limb absence, (2) were medically unwell, (3) were pregnant, and (4) if acupuncture use was cautioned [13].

Participants were randomly allocated to either receive usual care and acupuncture or usual care alone. A usual care comparator was chosen as the study was undertaken under the Medical Research Council guidelines for developing and evaluating complex interventions. Usual care included pharmacological medical intervention, physiotherapy, and occupational therapy. Acupuncture was provided by an NHS clinic colocated in the same building by one British Acupuncture Council-registered acupuncture practitioner (BSc (Hons) Acupuncture) with more than 15 years of clinical experience. Acupuncture was delivered pragmatically under the Traditional Chinese Medicine (TCM) paradigm. A protocol developed prior to the study, using Delphi consensus methodology was used to provide guidelines [14] and included:

- Using a combination of body and auricular acupuncture
- Treating the contralateral limb and possibly the ipsilateral limb
- Including auricular acupuncture points such as the *Shen Men* and the sympathetic and points corresponding to the lower limb
- Depending on the health of the tissue and the individual participant, needling around the stump
- Mirroring local and distal points by needling the opposite limb
- Including points on the lower back (taking a segmental approach to dermatomal pain)
- Including points such as LI4 + LR3, LR3, GV20, SP10, and also specified points according to participants’ specific symptoms
- Attempting to obtain *deqi*
- Retaining needles for 20–30 min

Treatment could include electroacupuncture or other adjunctive interventions including cupping, exercises, and lifestyle advice. All participants in the acupuncture group were allocated eight 1-h sessions (twice weekly for 4 weeks).

Outcome measures were completed at baseline, weekly for the duration of the trial and 1 month post completion of the study. The primary outcome measure was an 11-point numerical rating scale (NRS) capturing average PLP over the past week, using the anchors 0 meaning ‘no pain’ and 10 meaning ‘pain as bad as you can imagine’ [15]. Secondary outcome measures included; NRS capturing worst PLP over the past week, the Short Form McGill Pain Questionnaire 2 (SF-MPQ-2) [16], the EuroQol-5 dimensions, 5 levels (EQ-5D-5 L) questionnaire [17], the Hospital Anxiety and Depression Scale (HADS) [18], the Perceived Stress Scale 10-item (PSS-10) [19], the Insomnia Severity Index (ISI) [20], and a 7-point Patient Global Impression of Change (PGIC) scale ranging from 1 meaning ‘no change’ to 7 meaning ‘a great deal better’. Phrasing of the PGIC question was similar to the phrasing used by Hurst and Bolton [21] and stated ‘since being enrolled in this study how would you describe the change (if any) in activity limitations, symptoms, emotion, and overall quality of life in relation to your phantom limb pain?’ Feasibility-specific data were collected (Table 4) and post completion of the study, participants in the acupuncture group were interviewed. Interviews were conducted by the researcher who enrolled participants and collected outcome measures. Interviews were semistructured, audio-recorded, followed a topic guide and were transcribed verbatim.

No sample size calculation was undertaken but a sample of 20 was deemed adequate to inform on feasibility [22]. Interim safety and effectiveness were not formally evaluated but data were collected through participant interviews. Randomisation and allocation concealment was undertaken by a researcher not involved in the study using a computer-generated random numbers table. Randomisation was unstratified and balanced using a block size of 4 and allocation concealment was implemented using sequentially numbered opaque envelopes which were only opened once participants had been enrolled. The researcher collecting outcome measures and analysing the data enrolled the participants and was blinded to their allocation. Participants and acupuncture practitioners were not blinded.

Quantitative data analysis used an intention-to-treat approach and missing data were imputed using last observation carried forward. The intervention was discontinued after week 4 and this was chosen as the primary endpoint of the study (day 28). As this was a feasibility study no significance tests were performed and no hypothesis testing is reported. Raw change, the difference between mean baseline and subsequent scores was calculated for the NRS and considered meaningful/clinically significant when ≥1.80 [23]. Cohen’s *d* effect size was calculated using the calculation:

$$ d\kern0.5em =\kern0.5em {M}_1\hbox{--} {M}_2/\sigma, $$

where *M* = mean and *σ =* standard deviation, pooled using Cohen’s criteria: 0.2, small effect; 0.5, medium effect; and 0.8, large effect [24]. Framework Analysis [25] was used to analyse qualitative data. Specific steps were followed during data analysis including: familiarisation, coding, identifying an analytical framework, indexing, charting, and mapping/interpretation. All codes and themes were developed inductively during analysis of the data. Inferences were drawn from analysis of qualitative and quantitative findings. Meta-inferences were drawn through combining qualitative and quantitative findings using side-by-side comparison [26].

Using effect size data generated from this study and taking the assumption that a future study would: (1) use an 11-point NRS measuring average pain over the last week, (2) have normally distributed data, (3) use a two-tailed independent samples *T* test to compare acupuncture versus usual care, and (4) set power and level of significance/*α*-level at 0.8 and 0.05, respectively, enabling a sample size for a future definitive trial to be calculated [27]:

$$ n\kern0.5em =\kern0.5em \frac{2{\left({Z}_{\mathrm{a}}\kern0.5em +\kern0.5em {Z}_{1-\beta}\right)}^{2\sigma 2,}}{\varDelta^2} $$

where, *Z*_*a*_ = 1.96 [27], *Z*_*1-β*_ = 0.8416 [27], σ = standard deviation (estimated) and Δ = estimated effect size.

## Results

**Fig. 1:**
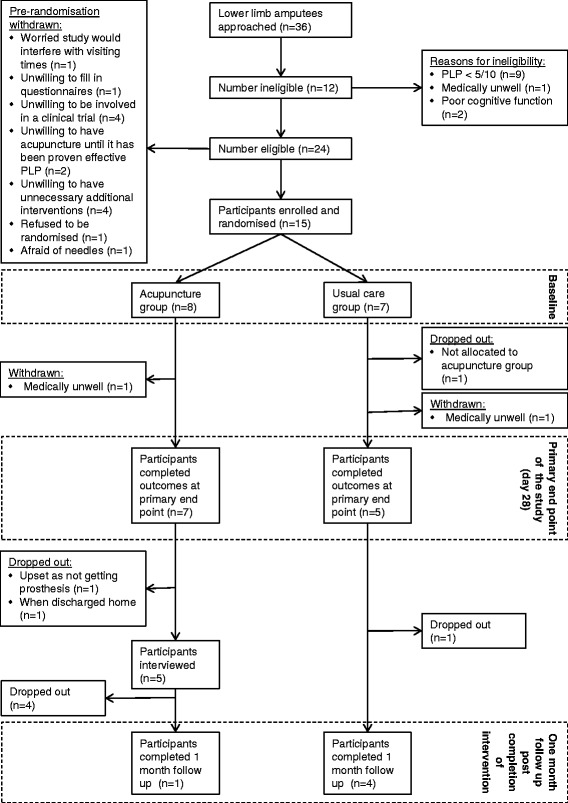
Participant flow through the trial

A total of 36 lower limb amputees were identified of whom 12 were ineligible. Of those eligible 9 refused to participate. A total of 15 participants were enrolled and their data were analysed within their originally assigned groups. Before the primary endpoint two were withdrawn due to being medically unwell and one dropped out having been randomised to usual care. A total of 12 participants completed outcomes at day 28, the primary endpoint of the study (seven in the acupuncture group and five in the usual care group). A total of 10 participants did not complete the 1-month follow-up questionnaire and 2 participants refused to be interviewed at the end of the study (Fig. 1).

Demographic details are presented in Table 1. Between groups there were differences in gender. In the acupuncture group six were men and in the usual care group five were women. In the acupuncture group the majority of participants were below-knee amputees, whereas in the usual care group the majority were above-knee amputees. Baseline primary and secondary outcome measure scores were similar between groups.

**Table 1:** Participant demographics

Participant demographics	Acupuncture group (*n* = 8)	Control group (*n* = 7)
Age mean (±95 % CI)	51.63 (40.38–62.87)	55.71 (40.17–71.26)
Gender *n* (%)		
Male	6 (75.00)	2 (28.57)
Female	2 (25.00 )	5 (71.43)
Ethnicity *n* (%)		
White British	7 (87.50)	4 (57.14)
Black Caribbean	1 (12.50)	1 (14.29)
Black African	0 (0.00)	1 (14.29)
White other	0 (0.00 )	1 (14.29)
Employment status *n* (%)		
Student	0 (0.00)	1 (14.29)
Unemployed	1 (12.50)	0 (0.00)
Sick leave	5 (62.50)	3 (42.86)
Retired	2 (25.00)	3 (42.86)
Time since amputation in days mean (±95 % CI)	25.63 (18.43–32.85)	29.43 (13.12–45.74)
Level of amputation *n* (%)		
Above-knee	2 (25.00 )	4 (57.14)
Below-knee	6 (75.00)	3 (42.86)
Reason for amputation *n* (%)		
Vascular	5 (62.50)	3 (42.86)
Trauma	2 (25.00)	2 (28.57)
Infection	0 (0.00)	1 (14.29)
Other	1 (12.50)	1 (14.29)
History of past amputations? *n* (%)		
Yes	2 (25.00)	1 (14.29)
No	6 (75.00)	6 (85.71)
General health *n* (%)		
Diabetes I	1 (12.50)	0 (0.00)
Diabetes II	3 (37.50)	2 (28.57)
Cancer	1 (12.50)	0 (0.00)
Osteoarthritis	1 (12.50)	0 (0.00)
Epilepsy	1 (12.50)	0 (0.00)
Nil	1 (12.50)	5 (71.43)
Mobility level *n* (%)		
Wheelchair user	8 (100.00)	7 (100.00)

*CI* confidence interval

### Quantitative findings

**Fig. 2:**
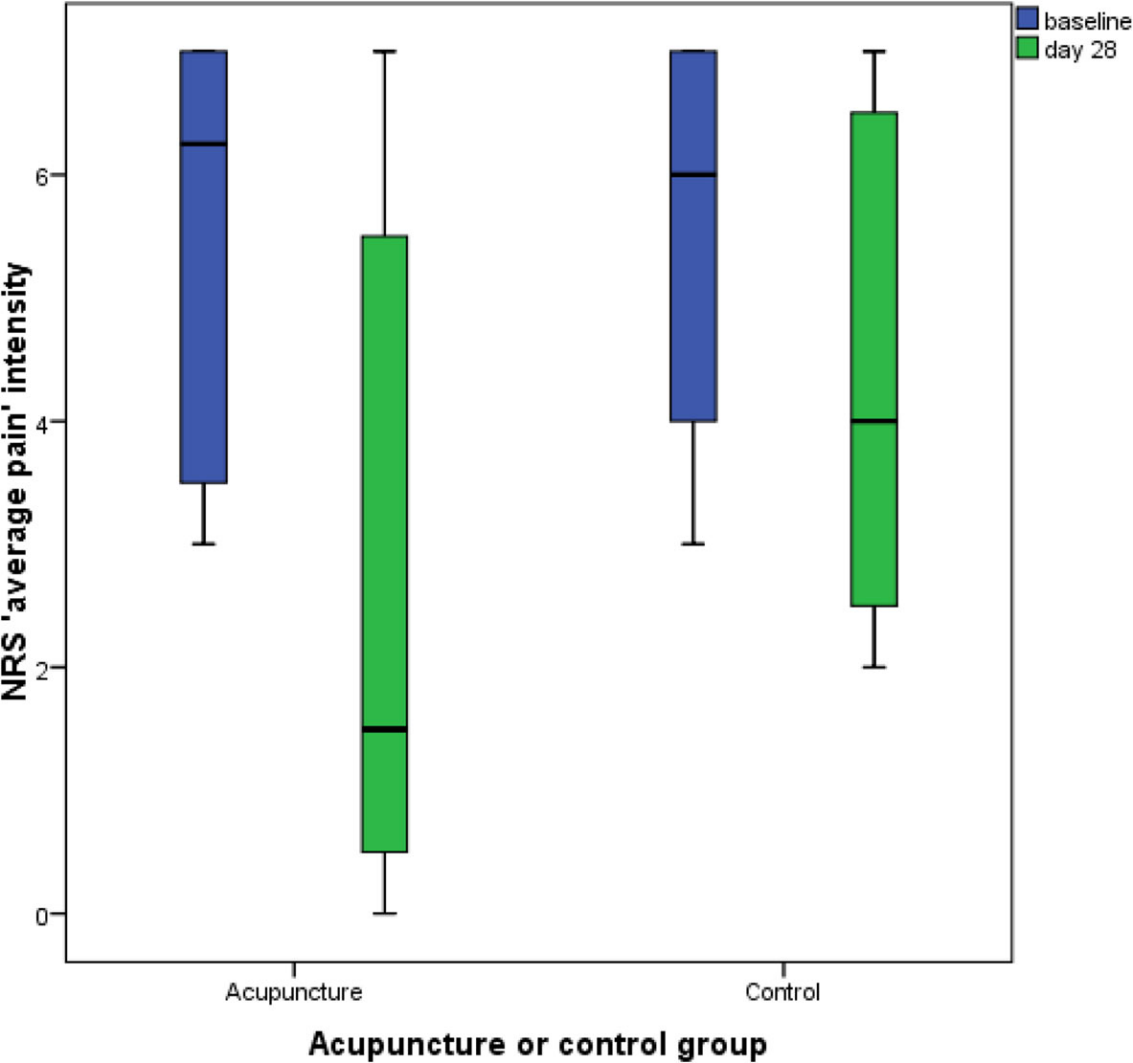
Box plot of ‘average pain’ intensity at baseline and day 28

In the acupuncture group mean average pain decreased from 5.44 to 2.75 and in the usual care group from 5.43 to 4.43 (Fig. 2). In the acupuncture group decrease in average pain was found to be clinically meaningful (raw change = 2.69), but not in the usual care group (raw change = 1.00). At day 28, a medium effect was found between groups (*d* = 0.64).

In the acupuncture group decrease in mean worst pain was found to be clinically meaningful (raw change = 4.00) but this was not so in the usual care group (raw change = 1.00). The SF-MPQ-2 identified a small effect between groups at day 28 (*d* = 0.46). Mean HADS anxiety and depression scores were normal throughout the study in both groups (score ≤7). As with the HADS, little change was observed in PSS-10 scores over the course of the study. Both groups at baseline had subthreshold insomnia (ISI score 8–14) which improved by day 28. Throughout the study EQ-5D-5 L scores were stable across dimensions. At the primary endpoint of the study the PGIC identified that participants in the acupuncture group rated themselves as ‘better’ whereas participants in the usual care group rated themselves as ‘a little better’. The datasets supporting these findings are included in Table 2.

**Table 2:** Summary statistics at baseline and day 28 expressed as mean and between-group effect sizes

Outcome measure	Group	Baseline mean (±95 % CI)	Between-group effect size (Cohen’s *d*)	Day 28 mean (±95 % CI)	Between-group effect size (Cohen’s *d*)
Number of participants who provided complete data	Acupuncture	8		7	
	Usual care	7		5	
NRS average pain	Acupuncture	5.44 (3.90–6.98)	0.00	2.75 (0.31–5.19)	0.64
	Usual care	5.43 (3.75–7.11)		4.43 (2.37–6.49)	
NRS worst pain	Acupuncture	8.00 (6.21–9.79)	0.38	4.00 (0.40–7.60)	0.69
	Usual care	7.29 (5.80–8.77)		6.29 (4.54–8.03)	
SF-MPQ-2	Acupuncture	2.55 (1.70–3.40)	0.21	1.06 (0.13–2.24)	0.46
	Usual care	2.85 (1.22–4.47)		1.89 (0.07–3.85)	
HADS anxiety	Acupuncture	6.38 (2.75–10.00)	0.29	5.25 (1.97–8.53)	0.10
	Usual care	5.29 (2.33–8.24)		4.86 (1.38–8.34)	
HADS depression	Acupuncture	6.63 (3.34–9.91)	0.35	0.35 5.75 (1.35–10.15)	0.12
	Usual care	5.14 (0.99–9.29		5.14 (0.99–9.29)	
PSS-10	Acupuncture	15.25 (10.90–19.60)	0.31	11.63 (5.43–17.82)	0.48
	Usual care	17.28 (10.11–24.46)		15.57 (7.35–23.79)	
ISI	Acupuncture	13.50 (5.96–21.04)	0.49	8.50 (1.65–15.35)	0.14
	Usual care	9.14 (0.93–17.35)		7.42 (0.61–14.24)	
EQ-5D-5 L mobility	Acupuncture	4.88 (4.58–5.17)	0.00	3.75 (2.88–4.62)	0.47
	Usual care	4.88 (4.58–5.17)		4.29 (3.13–5.45)	
EQ-5D-5 L self care	Acupuncture	1.75 (1.16–2.34)	0.24	1.63 (1.00–2.25)	0.09
	Usual care	1.57 (0.84–2.30)		1.57 (1.08–2.07)	
EQ-5D-5 L usual activities	Acupuncture	3.75 (2.68–4.82)	0.45	2.88 (2.18–3.57)	0.84
	Usual care	4.29 (3.26–5.31)		3.71 (2.69–4.74)	
EQ-5D-5 L pain-discomfort	Acupuncture	3.50 (2.87–4.13)	1.24	2.88 (2.18–3.57)	0.44
	Usual care	2.71 (2.26–3.17)		2.57 (2.08–3.07)	
EQ-5D-5 L anxiety/depression	Acupuncture	2.00 (1.11–2.89)	0.46	2.00 (1.23–2.77)	0.50
	Usual care	1.57 (0.84–2.30)		1.57 (0.84–2.30)	
EQ-5D-5 L health today	Acupuncture	63.13 (46.41–79.84)	0.21	74.63 (58.49–90.76)	0.15
	Usual care	67.14 (50.29–84.00)		77.14 (63.05–91.23)	
PGIC	Acupuncture			5.71 (4.23–7.20)	1.27
	Usual care			3.20 (0.37–6.03)	

*CI* confidence interval, *EQ-5D-5 L* EuroQol-5 dimensions, *HADS* Hospital Anxiety and Depression Scale, *ISI* Insomnia Severity Index, *NRS* numerical rating scale, *PGIC* Patient Global Impression of Change, *PSS-10* Perceived Stress Scale 10-item, *SF-MPQ-2* Short Form McGill Pain Questionnaire 2

### Qualitative findings

Six themes were identified through interviews with participants who received acupuncture (Table 3). Participants were initially sceptical and apprehensive about being involved in the trial, had low expectations of acupuncture and hoped to be randomised to usual care. However, these views changed; participants liked treatment (even if it was not physically needed) and found it relaxing. Electroacupuncture was considered beneficial and pleasant and receiving two treatments a week was considered acceptable though some participants found this tiring. Acupuncture was perceived to be effective at resolving or reducing PLP and other health problems and four to six treatments were needed for it to be effective. Acupuncture was not perceived to cause any adverse effects. The environment where the acupuncture was conducted was considered to affect the effectiveness of treatment.

**Table 3:** Acupuncture group participant quotes from semistructured interviews

Theme	Quote
Scepticism and lack of expectations	‘I was a bit worried about what it was all about. You said “acupuncture” and I said “I’m not keen on that”. And then I thought I’ll try it, I’ll try it out.’ (Interviewee 1) ‘Didn’t expect it to work. Very, very, very sceptical me, very. That’s how ignorant I was… I didn’t think it would work, I thought it was all nonsense.’ (Interviewee 4)
Being treated	‘It was so relaxing… and, um, I was just lying there and I could have quite easily gone to sleep! It was so relaxing, so sort of peaceful.’ (Interviewee 2)
Changes in phantom limb pain	‘I said “well, it’s good. It’s very good…” It works good and then last time she came she said “aren’t you going to have it?” I said “no, no, it’s got rid of that pain that was down there”.’ (Interviewee 1)
Factors affecting treatment	‘She (the acupuncturist) looked after you well and I think that was a lot of it, her personality and the way she treated you and everything.’ (Interviewee 2) ‘I think because of the environment it was being done in and the timing more than anything, I think it wasn’t really a positive thing. It might have been a different story in another setting, if I had more time around my schedule.’ (Interviewee 3)
Completing the outcome measures	(Re the SF-MPQ-2) ‘ A couple of the wordings were a bit weird. I didn’t get some of the words on the describe the pain, gruelling and what was the other one? There were a couple of them I didn’t understand what these words meant.’ (Interviewee 4)
A good experience	‘It’s been very positive… it’s been an extra benefit I would definitely say that… I couldn’t criticise anything to be perfectly honest.’ (Interviewee 5)

*SF-MPQ-2* Short Form McGill Pain Questionnaire 2

Completing outcome measures was considered acceptable and relevant, but the SF-MPQ-2 included words which some participants did not understand. Length of time and frequency of questionnaire completion was acceptable with only one participant thinking that they were given too often. Overall, being involved in the study was considered a good experience and acupuncture was perceived to be beneficial. Participant quotes are included in Table 3.

### Feasibility-specific findings

Recruitment was problematic, clinicians sometimes failed to identify suitable participants, the unit did not always run at full capacity and potential participants were often unwilling to be involved having just had a major amputation. Of those identified, 12 were ineligible for inclusion, mainly due to PLP being less than 5/10 in intensity and of the remainder *n* = 24, 62.50 % consented to be enrolled. Randomisation worked well with only one participant dropping out due to being randomised to usual care and all participants were treated in the group they were allocated into. Those enrolled reported being happy to be randomised to either acupuncture or usual care. Blinding was unsuccessful, with both participants and practitioners unintentionally informing the researcher which group they had been allocated to.

Participant compliance to the protocol was good [14]. The four participant deviations were due to: tiredness, forgetting appointments, appointments coinciding with another medical appointment, and not wanting further treatment as PLP had resolved. Practitioner adherence to the protocol was poor and no participant received all eight treatments (mean total number of treatments 5.14 (4.02–6.27)). Despite the protocol [14] advising using a combination of auricular and body acupuncture this was only given to one participant on two occasions. Both lower limbs were treated 66.67 % of the time whereas for the contralateral limb this was only 8.33 %. Needle retention time and adverse events were not reported. For the dataset on acupuncture points used by practitioners in this feasibility study, see Additional file 2 (Acupuncture points used by practitioners during the feasibility study.pdf).

Outcome measures were identified which would be appropriate for a definitive trial. The NRS, SF-MPQ-2, and PGIC captured change. Baseline HADS scores were normal and little change was observed in the PSS-10 and the EQ-5D-5 L suggesting that these outcomes may be inappropriate. The ISI may not be appropriate in an inpatient setting as anecdotally noise and medication affected sleep. Retention of participants up until the primary endpoint of the study was good, but at 1-month follow-up was poor.

A sample size for a future trial was calculated corresponding to an effect size of 0.64 and pooled standard deviation of 1.36. A total of 71 per group (142 in total) would be needed. According to findings from this feasibility study, the follow-up rate at 4 weeks was 80 %. Therefore, considering a 20 % dropout rate, 170 participants (85 per group) are recommended to detect a significant change in a two-armed, parallel-group randomised controlled trial comparing acupuncture and usual care as measured using an 11-point NRS measuring average pain at 4 weeks.

Using the criteria set a priori [12], as shown in Table 4 the study was found to be successful in relation to participants receiving the intended intervention, outcome measures being considered acceptable and appropriate and being completed at the primary endpoint of the study and the intervention being considered acceptable and appropriate for use in a definitive trial. The study was unsuccessful in relation to recruitment, practitioner adherence to the protocol, completion of outcome measures at 1-month follow-up and blinding.

**Table 4:** Success of feasibility study

A priori criteria	Findings	Objective met? (yes/no)
Recruitment rate was ≥2 participants per month fitting the eligibility criteria	Recruitment rate was 1.36 eligible participants per month	No
The study recruited ≥70 % of all eligible potential participants	62.50 % of all eligible participants were recruited	No
Of the participants recruited to acupuncture group ≥90 % received their first acupuncture treatment within 1 week of recruitment	All participants received their first acupuncture treatment within 1 week of recruitment	Yes
After randomisation and allocation ≥90 % of participants received treatment as initially intended	All participants received treatment as intended and the study protocol was considered acceptable	Yes
Of the participants recruited to acupuncture group ≥80 % received all 8 acupuncture treatments	No participants received all 8 treatments (mean total number of treatments 5.14 (4.02–6.27))	No
Of the participants recruited to usual care group ≤10 % dropped out of the study	One participant (14.29 %) of participants dropped out of the usual care group	No
At the primary endpoint of the study outcome measures were completed by ≥90 % of participants	100 % of participants still enrolled on the study completed all outcome measures by the primary endpoint of the study	Yes
At 1 month after completion of the study, outcome measures were completed by ≥60 % of participants	Outcome measures were completed by 5 participants (33.33 %)	No
Qualitative data identified that outcome measures were acceptable and appropriate, that questionnaires and rating scales were easy to complete and that outcome measures could be identified for use in a definitive trial	Outcome measures were considered acceptable, appropriate and easy to complete. The HADS, PSS-10, EQ-5D-5 L, and ISI may not be appropriate for use in a definitive trial	Yes
Qualitative data implied that acupuncture was an acceptable and effective intervention for treating PLP with or without other secondary symptoms	Acupuncture/electroacupuncture was considered acceptable. Acupuncture was perceived to be effective at treating both PLP and other secondary complaints	Yes
Data were collected on the primary outcome measure (NRS) and effect size was calculated to inform a sample size calculation for a larger trial	Considering a 20 % dropout rate, 170 participants are recommended to be recruited to detect a significant change in a two-armed, parallel-group randomised controlled trial comparing usual care and acupuncture as measured using an 11-point NRS measuring average pain at 4 weeks	Yes
Qualitative and quantitative data implied that the acupuncture protocol used in the feasibility study was appropriate for use in a definitive multicentred randomised controlled trial	Participants did not drop out of the acupuncture group suggesting that it was acceptable. Participants’ symptoms generally improved over 6 treatments suggesting that 8 treatments was adequate. Acupuncture and electroacupuncture were considered acceptable, effective, and relaxing	Yes
The researcher was not aware which group participants had been enrolled to 100 % of the time	Blinding was not successful and the researcher knew through both participants and clinical staff at the amputee unit their group allocation	No

*EQ-5D-5 L* EuroQol-5 dimensions, *HADS* Hospital Anxiety and Depression Scale, *ISI* Insomnia Severity Index, *NRS* numerical rating scale, *PSS-10* Perceived Stress Scale 10-item, *SF-MPQ-2* Short Form McGill Pain Questionnaire 2

## Discussion

The study did not meet its target of recruiting at least 2 participants per month or 20 participants in total. This is not unusual and other studies have also reported recruitment as being slower or more difficult than expected [28]. It has been suggested that clinical staff have limited time to undertake research activities [29] and this may have influenced the identification of potential participants. A future trial would need to ensure that trial centres allocated adequate time and personnel. Potential participants should be provided with some education about the intervention as a brief introduction may make participants less sceptical and more willing to consent. Recruitment could be enhanced by a multicentred approach. Intensity of PLP was a major barrier to recruitment. Although PLP can be severe, this may only be in approximately 30 % of amputees [30, 31] explaining why this inclusion criterion excluded nine participants. Future studies may consider lowering or excluding the severity of this criterion.

The study did not meet its target of recruiting at least 70 % of all eligible participants. However, this criterion was unrealistically high and 62.50 % of all eligible participants were recruited. Other CAM studies report a lower participation rate [32] and studies evaluating the effectiveness of interventions for treating PLP also report a lower participation rate [33]. This study may not have met its target recruitment rate because it was set unrealistically high. Participation rate was good, suggesting that a future trial would be possible.

Amputees have often undergone extensive unpleasant interventions prior to amputation, and this may partly explain the reason for those refusing to consent. The study site may not have been optimal for recruiting due to it being a busy unit providing rehabilitation care for those at a key life point. Although, overall, recruitment was good, future studies may benefit by including amputees who are not in an inpatient unit receiving multiple interventions at a key life point and by making the proposed intervention less intensive.

Blinding was unsuccessful. A future study may benefit from clearly including information on the participant information sheet about the necessity of blinding and should ensure that the outcome measures used are reliable and objective. Additionally, a future trial could use duplicate assessments of outcomes and report the level of agreement between assessors [34]. Also, different data analysts to data collectors could be used.

Establishing acceptability and compliance to an intervention is vital because if the intervention is unacceptable and participants are not compliant, the study will fail. This study suggested that acupuncture and usual care were acceptable and participants were compliant with the protocol. Unlike usual care alone, acupuncture did appear to be clinically effective at reducing pain intensity and the findings suggested a ‘meaningful change’ [23]. This is in keeping with results from case studies [11] and nonrandomised controlled trials [10]. Clinically meaningful change is important as this is relevant to patient care. Across a diverse patient group a change of 1.74 on an 11-point NRS has been associated with ‘much improved’ and a change of 2.76 ‘very much improved’ [35] suggesting that by the primary endpoint participants in the acupuncture group average pain was ‘much improved’ and worst pain was ‘very much improved’ but this was not so in the usual care group for either average or worst pain. In keeping with quantitative findings, qualitatively acupuncture was perceived to be effective at resolving symptoms. Findings from this study support the need for a definitive trial to determine effectiveness. As less than eight treatments may be effective this may be a more appropriate and cost-effective dosage. A usual care control should be used in future studies as it has the advantage of being safe (physicians make individualised treatment decisions about participant care) and, unlike efficacy trials, ensures that the intervention can claim to be superior to usual practice [36].

Unexpectedly, practitioners were found to not adhere to the acupuncture protocol. This lack of adherence may have been partly due to tensions between clinical and research workload [29] and also due to poor communication with the research team. This would need addressing before undertaking a future trial as lack of participants receiving the full intervention as intended could lead to reduced effectiveness, a decrease in study power and inappropriate conclusions [37]. Robiner [38] provides a table of adherence-enhancing strategies which could be used in a future trial, including: promoting collaboration and good communication between acupuncturists and research staff, providing feedback on adherence, promoting nonjudgemental discussion around adherence, and addressing adherence problems proactively.

Although adverse events were captured during semistructured interviews, practitioner compliance of capture of adverse effects was poor. This is not uncommon [39] but would need addressing before undertaking a definitive trial. Recommendations of capture of adverse events include capturing the frequency, incidence, timing, and severity of each event [40]. A future study may benefit from giving practitioners a log book designed to capture this information.

The study identified appropriate outcome measures which could be used in a future trial. However, as the SF-MPQ-2 included some terminology which was not understood, an alternative outcome measure may be more appropriate such as the neuropathic pain scale, the neuropathic pain symptom inventory, or the Pain Quality Assessment Scale [41, 42]. Although participants adhered to completing outcome measures, this was not sustained post the primary endpoint of the study. This lack of long-term retention needs addressing as poor retention has implications for statistical power and the internal and external validity of a study [43]. Strategies could be implemented such as including a follow-up contact, prenotification reminders, and mentioning an obligation to respond [44]. In randomised controlled trials offering and giving small monetary incentives has been found to be successful in improving response [43]. However, lower limb amputees tend to be a frail population and long-term survival post amputation is poor. By 1 year post amputation almost half (44 %) of lower limb amputees will have died and by 5 years 77 % [45]. Additionally, major amputations are associated with high morbidity and complication rates. This would need to be taken into consideration when designing a definitive trial.

### Limitations

This study did not consider the effect of attention on symptoms and did not include a control that mimicked the theoretically inactive elements but not the active elements of acupuncture. Further research needs to be carried out to identify optimal dosage, which aspects of acupuncture intervention causes change and whether environmental factors affect outcomes. The study did not recruit the number of participants it initially aimed to recruit and the quantitative findings reported in this study should be interpreted with caution. Only one practitioner was involved in this study and as differences in effectiveness are known to occur with different practitioners [46] future studies would benefit from the use of multiple practitioners. Practitioners did not adhere to the acupuncture protocol and participants were not offered eight treatments, making it difficult to determine the effectiveness of the protocol. Two participants in the acupuncture group were not interviewed and data saturation of qualitative data cannot be assumed.

## Conclusions

The study provides novel data on the feasibility of conducting a randomised controlled trial to establish the effectiveness of acupuncture for treating lower limb amputees with PLP. The study identified that acupuncture may cause clinically meaningful change. The protocol used in this study was acceptable and data on effect size were generated allowing for a sample size calculation. Areas which would need addressing prior to undertaking a definitive trial were identified.

## Additional files

Additional file 1: CONSORT 2010 checklist of information to include when reporting a randomised trial. (PDF 144 kb)
Additional file 2: Acupuncture points used by practitioners during the feasibility study. (PDF 84 kb)

## Abbreviations

EQ-5D-5 L: EuroQol-5 dimensions, 5 levels questionnaire
HADS: Hospital Anxiety and Depression Scale
ISI: Insomnia Severity Index
PGIC: Patient Global Impression of Change
PLP: Phantom limb pain
PSS-10: Perceived Stress Scale 10-item
SF-MPQ-2: Short Form McGill Pain Questionnaire 2
TCM: Traditional Chinese medicine
