# Supplementary material for: Acupuncture for the treatment of phantom limb syndrome in lower limb amputees: a randomised controlled feasibility study
Source: Trials. 2016 Oct 25;17:519. doi: 10.1186/s13063-016-1639-z (PMC5080724; doi:10.1186/s13063-016-1639-z)
Supplement: Additional file 2: — Acupuncture points used by practitioners during the feasibility study. (PDF 84 kb) [file 13063_2016_1639_MOESM2_ESM.pdf]

Acupuncture points used by practitioners during the feasibility study

Residual limb	F	Contralateral limb	F	Body points	F	Points on Lx	F	Auricular acupuncture	Upper limb points to treat lower limb	F
Sp 8	2	Sp 3	1	Yintang	1	Bl 23	5	Yes X 2*	LI 2	1
Sp 9	9	Sp 4	9	Du 20	1	Bl 24	3		LI 4	4
Sp 10	20	Sp 5	18	LI4	1	Bl 25	5		LI 5	2
Sp 11	1	Sp 6	17			Bl 26	5		LI 6	1
		Sp 9	17			Bl 27	2		LI 10	3
St 32	1	Sp 10	11			Bl 50	1		LI 11	4
St 33	1					Bl 52	1			
St 34	16	St 34	2						Lu 9	1
St 35	16	St 35	7			Du 4	3			
St 36	8	St 36	8						PC6	1
St 37	1	St 41	11			GB 29	2		PC7	1
		St 42	2			GB 30	2			
GB 34	4	St44	5						TB 5	1
GB 35	1					Huatuojiaji	1			
		GB 34	5							
LR 8	4	GB 39	5							
		GB 40	6							
Heding	2	GB 42	1							
Xiyan	14	GB 43	1							
Ashi point	1									
		LR2	4							
		LR3	8							
		LR8	4							
		Bl 57	1							
		Bl 58	4							
		Kid 3	7							
		Kid 5	3							
		Kid 6	2							
		Kid 7	2							
		Kid 10	1							
		Xiyan	4							
		Ashi Points	2							
		Heding	2							
		Bafeng	1							
**Other:**<br>Cupping Tx, F X 1<br>Points for the shoulder, F X 9										

Key: F, frequency; *, acupuncture points not recorded; Tx, thoracic area.
Points for the shoulder: LI 14, LI 15, LI 16, TB 13, TB 14 SI 14, SI 15, Jianqian, ashi points.
